# A 3-miRNA Risk Scoring Signature in Early Diabetic Retinopathy

**DOI:** 10.3390/jcm12051777

**Published:** 2023-02-23

**Authors:** Jiali Wu, Ke Shi, Fang Zhang, Xiaodong Sun

**Affiliations:** 1 Department of Ophthalmology, Shanghai General Hospital, School of Medicine, Shanghai Jiao Tong University, Shanghai 200080, China; 2 Shanghai Engineering Center for Visual Science and Photomedicine, Shanghai 200080, China

**Keywords:** diabetic retinopathy, differentially expressed gene, miRNA, risk score signature

## Abstract

Purpose: The aim of our study was to investigate a comprehensive profile of streptozotocin (STZ)-induced early diabetic retinopathy (DR) mice to identify a risk scoring signature based on micorRNAs (miRNAs) for early DR diagnosis. Methods: RNA sequencing was performed to obtain the gene expression profile of retinal pigment epithelium (RPE) in early STZ-induced mice. Differentially expressed genes (DEGs) were determined with log2|fold change (FC)| > 1 and *p* value < 0.05. Functional analysis was carried out based on gene ontology (GO), Kyoto Encyclopedia of Genes and Genomes (KEGG) pathway enrichment analysis and the protein–protein interaction (PPI) network. We predicted the potential miRNAs via online tools and ROC curves were then conducted. Three potential miRNAs with AUC > 0.7 were explored via public datasets and a formula was further established to evaluate DR severity. Results: In total, 298 DEGs (200 up-regulating and 98 down-regulating) were obtained through RNA sequencing. Hsa-miR-26a-5p, hsa-miR-129-2-3p and hsa-miR-217 were three predicted miRNAs with AUC > 0.7, suggesting their potential to distinguish healthy controls from early DR. The formula of DR severity score = 19.257 − 0.004 × hsa-miR-217 + 5.09 × 10^−5^ × hsa-miR-26a-5p − 0.003 × hsa-miR-129-2-3p was established based on regression analysis. Conclusions: In the present study, we investigated the candidate genes and molecular mechanisms based on RPE sequencing in early DR mice models. Hsa-miR-26a-5p, hsa-miR-129-2-3p and hsa-miR-217 could work as biomarkers for early DR diagnosis and DR severity prediction, which was beneficial for DR early intervention and treatment.

## 1. Introduction

Diabetic retinopathy (DR) is one of the leading causes of vision impairment and even blindness in the working-age population. It is estimated that there are more than 90 million DR patients worldwide [1]. Clinically, DR is divided into two stages: non-proliferative diabetic retinopathy (NPDR) and proliferative diabetic retinopathy (PDR). NPDR is the early stage of DR without severe symptoms; therefore, most patients are not vigilant about being diagnosed. However, many pathological alterations, including increased vascular permeability and capillary occlusion, begin to occur at this stage [2]. Once NPDR progresses to PDR, vision dramatically decreases, posing a massive medical and economic burden on both patients and society. Therefore, a comprehensive study of early-stage DR is indispensable for early diagnosis and timely intervention.

Traditionally, DR was classified as a microvascular complication of diabetes. With the development of modern technologies, researchers gradually realized that DR not only affects the retina, but also influences the RPE and neuronal units in the early stage [3,4]. RPE has vital physiological functions, including the formation of the outer blood retinal barrier (oBRB), transportation of nutrients to photoreceptors (PRs), absorption of scattered light, recycling of retinoid and phagocytosis of shed PR outer segment membrane [5]. Therefore, depicting the gene profile of RPE and studying their function could help enrich the understanding of early DR and further develop novel diagnosis and treatment strategies [6,7].

In our study, we carried out the RNA sequencing for RPEs from early DR mice models. Potential miRNAs were predicted based on the DEGs in RPE and subsequently validated via a public dataset. A formula was then constructed to score the DR risk with potential miRNAs.

## 2. Materials and Methods

### 2.1. Animal Model of STZ-Induced DR

Male C57BL/6J mice aged 6–8 weeks were purchased from Shanghai Laboratory Animal Center. Intraperitoneal injection of 55 mg/kg of STZ was performed for 5 consecutive days to induce diabetes while PBS was utilized as a control. Blood glucose levels were tested via tail vein blood after one week, and animals with blood glucose concentrations of ≥ 200 mg/dL were considered to be successful models. Optical coherent tomography (OCT) was performed to observe the retinal structure before sacrifice. All animal experiments were approved by the Ethics Committee of Shanghai Jiao Tong University, China, and were in accordance with the requirements of the Association for Research in Vision and Ophthalmology Statement for the Use of Animals in Ophthalmic and Vision Research. The approval number of Animal Ethical Committee is 2019AW055.

### 2.2. RNA Sequencing

Gene expression profile in the RPEs was identified by RNA sequencing. RPE–choroid complexes were separated along the corneal limbus and transferred to 1.5 mL tubes with RNA protect Cell Reagent (QIAGEN, Dusseldorf, Germany) to enrich RPE cells. TRIzol (Invitrogen, Carlsbad, CA, USA) was subsequently used to extract total RNA. The genes with log2|fold change (FC)| > 1 and *p* values < 0.05 were defined as differentially expressed genes (DEGs) and visualized by volcano plots and heatmaps using R3.5.0 (R Foundation for Statistical Computing, Vienna, Austria). The expression pattern was available in Table S1.

Gene ontology (GO) and Kyoto Encyclopedia of Genes and Genomes (KEGG) pathway enrichment analysis was performed using Metascape (https://metascape.org/gp/index.html#/main/step1 accessed on 9 December 2022). GO, KEGG and reactome terms with *p* values < 0.05 were presented.

### 2.3. Protein–Protein Interaction (PPI) Network Construction

The Search Tool for the Retrieval of Interacting Genes/Proteins (STRING) (https://string-db.org/ accessed on 9 December 2022) was utilized to evaluate the protein–protein interaction (PPI) of DEGs. Cytospace (version 3.8.2) was further applied to construct the network with Cytohubba plug-in unit and the genes were ranked according to MCC algorithm. The redder gene represented the higher rank.

### 2.4. Construction of miRNA–mRNA Network

miRNet (https://www.mirnet.ca/ accessed on 9 December 2022) tool was utilized to predict miRNAs targeting DEGs, and Cytospace (version 3.8.2) was used to visualize the network.

### 2.5. Electroretinogram (ERG) Recording

Electroretinogram (ERG) recording was carried out with a scotopic Ganzfeld ERG system (Phoenix Research Labs, New York, NY, USA). The mice were anesthetized by intraperitoneally injecting 1.5% sodium pentobarbital (100 μL/20 g) and the pupils were dilated with tropicamide after dark adaptation overnight. The reference needle electrode was placed behind the ears while the ground one was plunged into the tail. As described below, the ERG was measured with four different stimulus intensities, 1.0, 2.0, 3.0 and 4.0 log cd s/m^2^ with intermittent intervals of 10, 20, 20 and 30 s. A-wave and B-wave values were recorded and analyzed.

### 2.6. Hematoxylin and Eosin (H & E) Histological Staining of Eyeballs

Eyeballs were enucleated and fixed in eyeball fix solution (Servicebio, Wuhan, China) after sacrifice. After wax embedding, eyeball cross sections were prepared (5 μm). The slides were stained in Hematoxylin solution for 5 min, followed by Hematoxylin differentiation solution and water rinse. Then, the slides were treated with Hematoxylin Scott Tap Bluing and rinsed with water again. Lastly, the slides were dehydrated and sealed with neutral gum.

### 2.7. Raw Data Acquisition

Raw data of the public datasets utilized in our study were acquired from Gene Expression Omnibus (GEO, https://www.ncbi.nlm.nih.gov/geo/ accessed on 9 December 2022). GSE1603086, based on GPL20301 platform, was a smallRNA profile consisting of retina samples from 43 donors. GSE1409597 was an miRNA profile with three different biological fluids, including aqueous humor, vitreous and plasma from 27 patients, which was established on GPL16384 platform.

### 2.8. Statistical Analysis

Data were shown as the mean ± SD. All the experiments were performed for at least three biological replicates and differences between the two groups were analyzed by a Student’s *t*-test. Linear regression analysis was used for analyzing relationships among miRNAs and DR severity. *p* values < 0.05 were considered statistically significant. Statistical analysis in our study was performed with SPSS Statistics 26 (IBM, Armonk, NY, USA).

## 3. Results

### 3.1. Identification of DEGs in Diabetic RPE via RNA Sequencing

**Figure 1:**
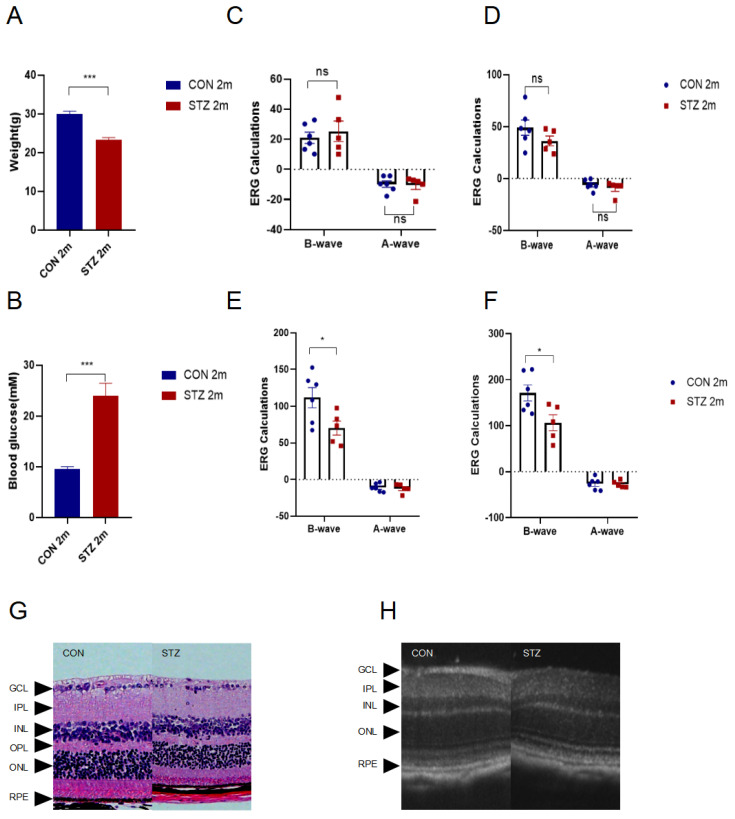
Establishment of DR mice models by intraperitoneal injection with STZ (*n* = 3). (**A**,**B**). Two months later, weight and blood glucose of mice were measured. (**C**–**F**). ERG recordings of flash 1, 2, 3, 4 in DR mice models. Bar graphs showed mean ± SD. *p*-values: Student’s *t*-test. (*** *p* < 0.001, *p* < 0.01, * *p* < 0.05, ns *p* > 0.05). (**G**). H & E staining of eyeball histology. (**H**). Eyeball histology showed by OCT in vivo. Asterisks point to different layers of retina. STZ: Streptozotocin; ns: not significant; GCL: Ganglion cell layer; IPL: Inner plexiform layer; INL: Inner nuclear layer; OPL: Outer plexiform layer; ONL: Outer nuclear layer; RPE: Retina pigment epithelium.

To our knowledge, there are no published sequencing data on RPEs in STZ-induced early DR models yet. Therefore, we established early DR mice models by STZ intraperitoneal injections. After 2-month STZ induction, diabetic mice showed lighter weight and higher blood glucose than control ones (Figure 1A,B). Electroretinograms (ERGs) were carried out to evaluate functional alterations. A wave of ERG represented the function of photoreceptors, while B wave indicated the function of the outer retina. ERG was taken with four different stimulus intensities, 1.0, 2.0, 3.0 and 4.0 log cd s/m^2^ with intermittent times of 10, 20, 20 and 30 s. As the stimulus increased, amplitudes gradually increased. As shown in the recordings, amplitudes of the B wave declined significantly in early diabetic mice while A waves showed no significant change. This finding suggested detectable functional damage to the retina instead of RPE at this stage (Figure 1C–F). Morphology changes were evaluated via H & E staining, from which we could observe a slight decrease in the outer nuclear layer. This thinning trend was consistent with that in OCT in vivo, both demonstrating retinal alterations in STZ-induced early diabetic models (Figure 1G,H).

**Figure 2:**
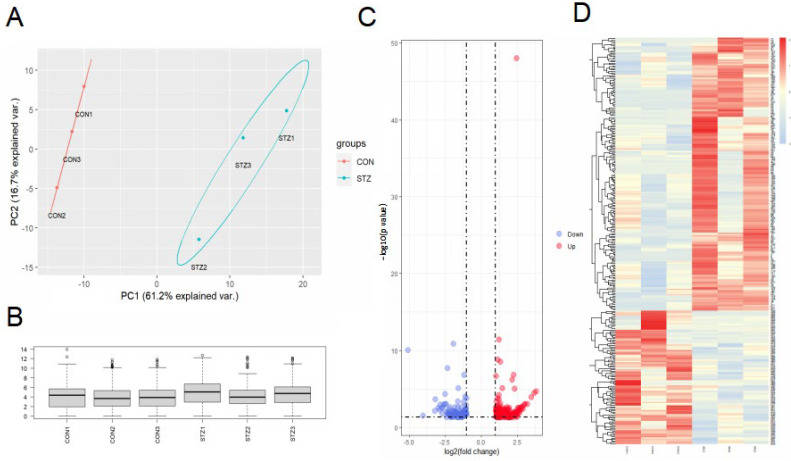
Identification of DEGs in the RNA sequencing of early DR mice models. (**A**). PCA blot for the repeatability of RNA sequencing. (**B**). Boxplot for the quality of RNA sequencing. (**C**) Volcano plot of the expression of DEGs; red nodes represent up-regulating genes while blue nodes represent down-regulating ones. (**D**). Heatmap of the expression of DEGs, red color represents higher expression while blue color represents lower expression.

We further performed RNA sequencing with STZ-induced early diabetic models, in an attempt to demonstrate a comprehensive gene profile (*n* = 3). PCA plot showed the reproducibility of our data within two groups (Figure 2A). Further, results of the sequencing were relatively uniform, seen by a distribution boxplot (Figure 2B). Genes with log2|FoldChange| > 1 and *p* value < 0.05 were identified as RPE-DEGs in our study. A total of 200 up-regulating and 98 down-regulating DEGs are shown by the volcano plot and heatmap (Figure 2C,D).

### 3.2. Pathway and Function Enrichment Analysis of RPE-DEGs

**Figure 3:**
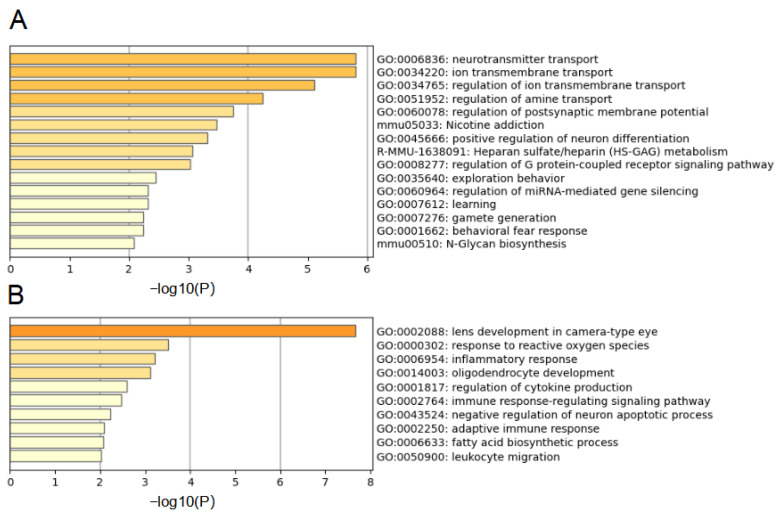
GO and KEGG pathway analysis of DEGs. (**A**). GO and KEGG pathway analysis for up-regulating DEGs. (**B**). GO and KEGG pathway analysis for down-regulating DEGs.

Metascape tool was utilized to carry out GO and KEGG analysis for RPE-DEGs. Neurotransmitter transport, ion transmembrane transport and regulation of amine transport were clustered in GO analysis for up-regulating DEGs (Figure 3A). Nicotine addiction, neuroactive ligand–receptor interaction, heparan sulfate metabolism and N-Glycan biosynthesis were enriched in KEGG pathways. Transmission across chemical synapses, neuronal system, neurotransmitter receptors and postsynaptic signal transmission and glycosaminoglycan metabolism were the top enriched reactome gene sets. When analyzing down-regulating genes, response to reactive oxygen species, inflammatory responses, regulation of cytokine production and fatty acid biosynthetic process were significantly enriched (Figure 3B).

**Figure 4:**
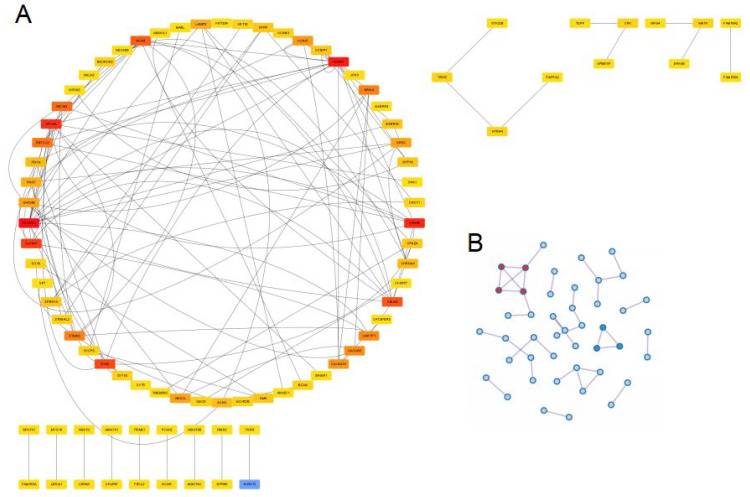

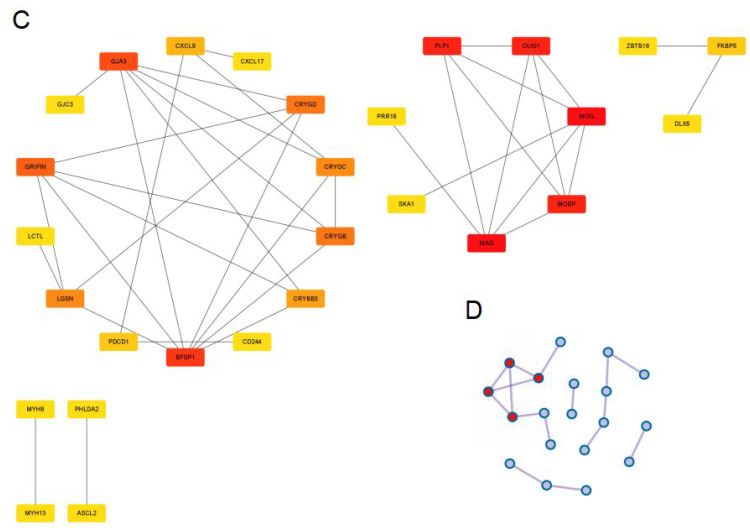
PPI network of DEGs. (**A**). PPI network of up-regulating DEGs;The redder gene represented the higher rank. (**B**). Densely connected gene sets in up-regulating DEGs. (**C**). PPI network of down-regulating DEGs. (**D**). Densely connected gene sets in down-regulating DEGs.

**Figure 5:**
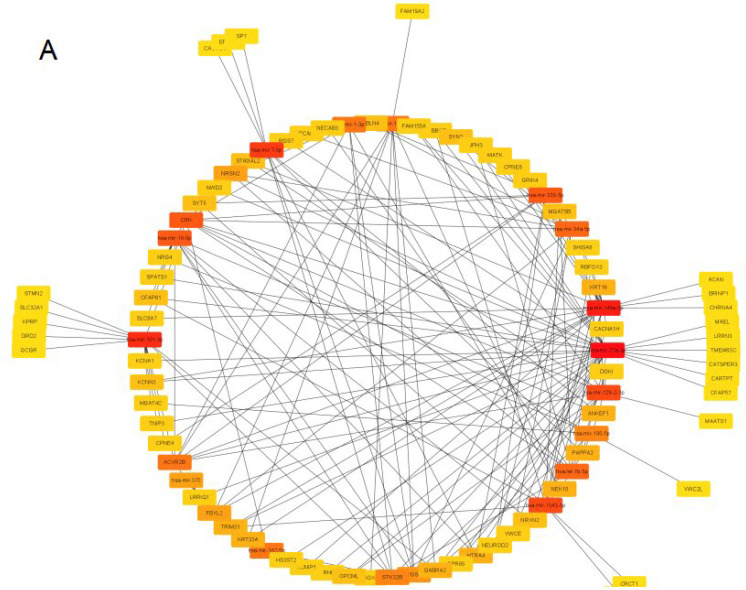

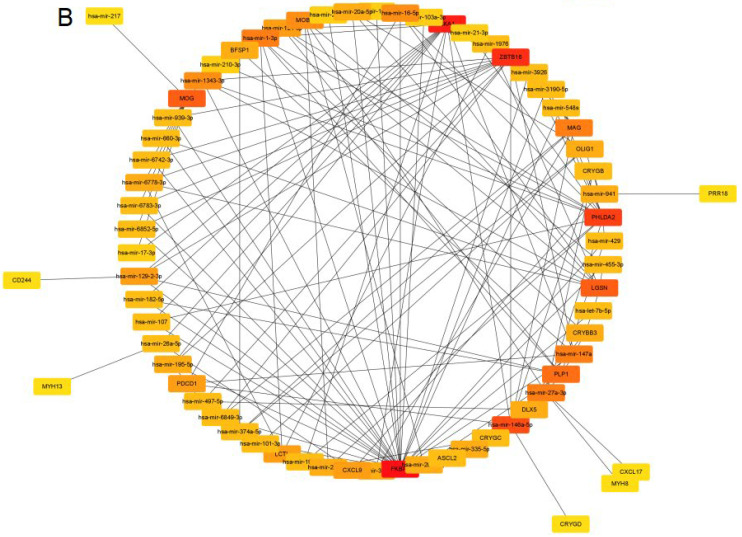
Construction of miRNA–mRNA networks. (**A**). miRNA–mRNA network for up-regulating DEGs with 15 miRNAs. (**B**). miRNA–mRNA network for down-regulating DEGs with 43 miRNAs.

The PPI networks were constructed for up-regulating and down-regulating DEGs separately via STRING database and further Cytoscape software. For the up-regulating DEGs, in total, 88 genes were identified. SLC32A1, KCNA1, CPNE6, OPCML, SLC6A7, SYN2, CBLN4, NCAN, NRXN2 and RBFOX3 were the top 10 genes ranked by the MCC method (Figure 4A). The Molecular Complex Detection (MCODE) algorithm was utilized to identify densely connected network components and 14 gene lists were gathered (Figure 4B). As for the down-regulating DEGs, MAG, MOG, MOBP, 0LIG1, PLP1, BFSP1, GJA3, GRIFIN, CRYGD and CRYGB were tagged as the top ten genes among 28 genes ranked (Figure 4C) and 5 individual gene sets were identified via the MCODE algorithm (Figure 4D).

### 3.3. Construction of miRNA–mRNA Regulatory Network

To further investigate the regulatory profile of these DEGs, we predicted and constructed miRNA–mRNA networks using the miRNet tool. In an attempt to search for the most promising miRNAs, we set the threshold of miRNAs targeting to at least 10% of DEGs. Thus, 15 miRNAs were mined targeting at least 9 up-regulating DEGs (Figure 5A) while 43 miRNAs were identified targeting at least 3 down-regulating DEGs (Figure 5B). miRNA–mRNA networks were further formed by Cytoscape. Among them, hsa-mir-27a-3p had potential to sponge with 37 up-regulating genes, and hsa-mir-146a-5p was the top miRNA, with the ability to target 14 down-regulating DEGs.

### 3.4. Confirmation of Potential miRNAs by GSE160308 and GSE140959

**Figure 6:**
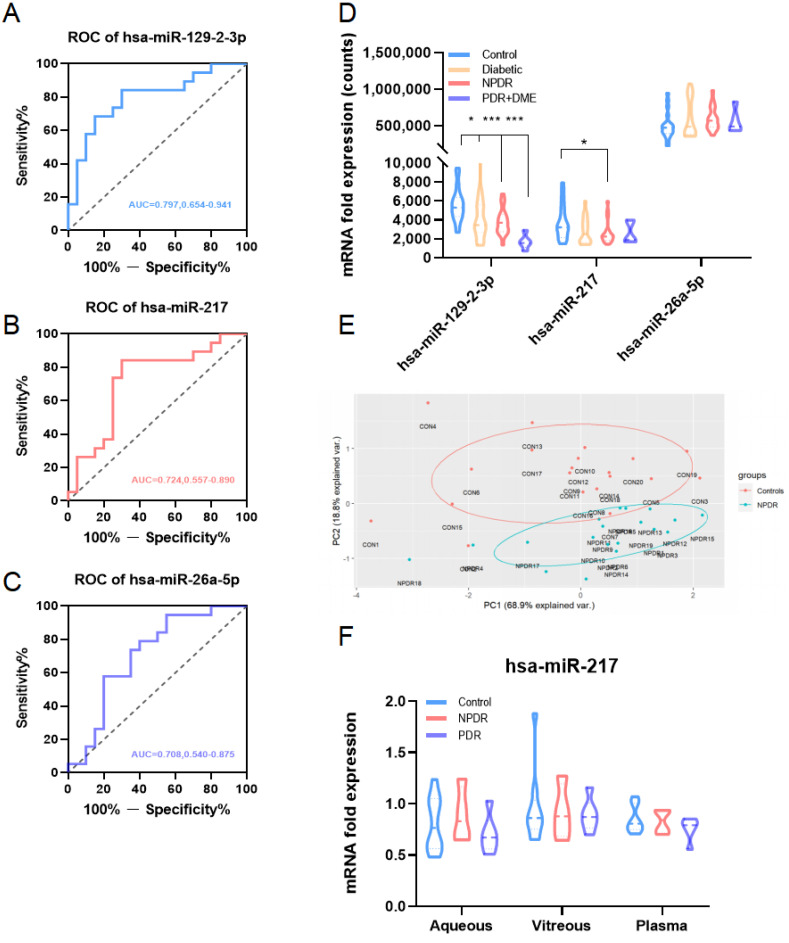
Validation of potential miRNAs by GSE160308. (**A**–**C**). ROC curves of three miRNAs with AUC > 0.7. (**D**). Expression level of miR-129-2-3p, miR-217 and miR-26a-5p in different groups of samples. Comparison of gene expression between two groups was performed by the Student’s *t*-test (mean ± SD, *** *p* < 0.001, *p* < 0.01, * *p* < 0.05). (**E**). PCA plot of healthy controls and NPDR patients by three miRNAs. (**F**). Expression level of miR-217 in different samples from DR patients.

After establishing the candidate miRNAs in early DR mice models, we further explored them in early-stage DR patient samples. GSE160308 was made up of 20 healthy control samples, 20 samples from diabetic patients without ocular manifestations, 19 non-proliferative DR patient samples and 5 DME patient samples. ROC curve analysis was performed for potential miRNAs. AUC > 0.7 was set as a meaningful cut-off value and AUC values of miR-129-2-3p (AUC = 0.797, 95%CI 0.654–0.941), miR-217 (AUC = 0.724, 95%CI 0.557–0.890) and miR-26a-5p (AUC = 0.708, 95%CI 0.540–0.875) were higher than 0.7, suggesting that they had potential to distinguish healthy controls from NPDR patients (Figure 6A–C). What’s more, miR-129-2-3p and miR-217 decreased along with the severity of DR while miR-26a-5p had an increasing trend (Figure 6D). The PCA plot also demonstrated that these three miRNAs could well distinguish NPDR from healthy controls (Figure 6E). GSE140959 was included for verification and miR-217 expression in this dataset also represented a decreasing tendency in aqueous, vitreous and plasma (Figure 6F). Association of these three miRNAs and severity of DR were further analyzed by regression analysis (Table 1). Results demonstrated that miR-129-2-3p (B = −0.003, β = −0.37, *p* = 0.002) and miR-217 (B = −0.004, β = −0.325, *p* = 0.028) were negatively related to DR progression. miR-26a-5p (B = 5.09 × 10^−5^, β = 0.584, *p* = 0.000) was positively associated with the risk of DR. This linear association could be calculated by: DR severity score = 19.257 − 0.004 × hsa-miR-217 + 5.09 × 10^−5^ × hsa-miR-26a-5p − 0.003 × hsa-miR-129-2-3p.

## 4. Discussion

DR is one of the leading causes of vision impairment in the working-age population. As a complex and multifactorial disorder, current therapies for DR, such as laser photocoagulation and anti-vascular endothelial growth factor injection, are not effective for all patients 4. The progression of NPDR to PDR is a vision-threatening turning point, and it is also the key point physicians should consider. By analyzing the gene expressions that change significantly in early DR, we predicted and constructed an miRNA-based risk signature for early DR diagnosis and therapy.

The role of RPE in DR is not well studied currently. RPE was a metabolically active tissue responsible for glucose trans-epithelial transport into the outer retina via GLUT1. Glucose was then utilized for synthesis of phospholipids via tricarboxylic acid cycle and oxidative phosphorylation. Therefore, RPE functioned as a bridge between choroid and photoreceptors to efficiently utilize glucose [8]. Diabetes not only disrupted RPE structure but also hampered the function of RPE cells. To the best of our knowledge, no sequencing study for RPE in diabetic mice has been reported yet. Therefore, we carried out RNA sequencing to investigate the gene profile in RRE cells.

Further, 200 up-regulating and 98 down-regulating DEGs were identified in our study. Functional analysis suggested that up-regulating DEGs played a vital role in neurotransmitter transport, ion transmembrane transport and regulation of amine transport. RPE was able to transport iron and iron was a necessary component in biological processes. However, excessive iron contributed to various pathological events, such as oxidative stress and lipid peroxidation. Iron accumulation was detected in postmorten human diabetic patients. With an HFE knockout (KO) mice model of genetic iron overload, researchers found that iron overload during diabetes exacerbated DR progression [9]. Excessive intracellular iron could be a fuse of ferroptosis, which was featured with mitochondrial atrophy and mitochondrial cristae structure change. In human retinal pigment epithelial (ARPE 19) cells treated with high glucose, intracellular ferrous iron increased and ferroptosis took place [10]. Neural deficits involving the GABA signaling pathway have been detected. GABA increases in the vitreous of PDR patients [11,12]. Hyperglycemia interferes with GABA signaling in the inner retina and rod-bipolar cells. It directly influences the GABA ρ subunit composition of GABAC receptors on retinal neurons [12,13,14,15]. When the GABAB receptor is activated, it can alleviate apoptosis and oxidative stress in neuronal cells via the PI3K-Akt signaling pathway in Alzheimer’s disease [16].

We further studied potential miRNAs targeting DEGs via the miRNet online tool and dozens of miRNAs were established. The public dataset GSE160308 was utilized for miRNA ROC curve exploration. Hsa-miR-217, hsa-miR-26a-5p and hsa-miR-129-2-3p were identified with AUC > 0.7, suggesting their potential to diagnose early DR. What’s more, miR-129-2-3p and miR-217 decreased along with the severity of DR while miR-26a-5p had an increasing trend. GSE140959 was then included and only one of three miRNAs, miR-217, was detected. Expression of miR-217 in this dataset represented a decreasing tendency in aqueous, vitreous and plasma of proliferative DR patients, which was consistent with that in GSE160308. All results suggested the vital roles of these three miRNAs in early DR.

miR-26a-5p was reported as a circulating biomarker for early-stage retinal neurodegeneration via plasma RNA sequencing from NPDR patients [17]. Mechanically, miR-26a-5p delayed thinning of neuroretinal layers by regulating PTEN expression. Further, miR-26a-5p up-regulation significantly decreased IL-1beta, NF-kapaB and VEGF expression [18]. Effects of miRNA-217 were investigated in high-glucose-treated ARPE-19 cells. miR-217 down-regulation augmented cell viability and alleviated cell apoptosis by sponging SIRT1. In addition, miR-217 inhibitor significantly reduced the expression of IL-1beta, IL-6 and tumor necrosis factor α [19]. miR-129-2-3p has not been reported in DR yet, but it was found to be beneficial to diabetic wound healing. Overexpression of miR-129-2-3p could accelerate wound healing by regulating inflammation and apoptosis directly [20]. Notably, the functions of most miRNAs we predicted have not yet been thoroughly elucidated, especially in early DR. Thus, further investigations are meaningful for prospective value validation.

In our results, these three miRNAs were all dis-regulated in DR. ROC curve analysis suggested their potential as early biomarkers for DR. What’s more, we found that the expression of three miRNAs was negatively or positively correlated with DR severity. Therefore, we further carried out regression analysis for them. miR-129-2-3p (B = −0.003, β = −0.37, *p* = 0.002) and miR-217 (B = −0.004, β = −0.325, *p* = 0.028) were negatively related to DR progression. miR-26a-5p (B = 5.09 × 10^−5^, β = 0.584, *p* = 0.000) was positively associated with DR severity. DR severity score could then be calculated by this three-miRNA risk scoring signature (F = 9.066, R = 0.516) as 19.257 − 0.004 * hsa-miR-217 + 5.09 × 10^−5^ * hsa-miR-26a-5p − 0.003 * hsa-miR-129-2-3p.

## 5. Conclusions

Our study identified a comprehensive gene profile in early diabetic mice models via RNA sequencing analysis, which expanded our understanding of pathological processes in DR. A three-miRNA risk scoring signature was further established for early diagnosis of DR. Further studies are warranted to explore and extend our findings for potential diagnosis and therapy development.

## Figures and Tables

**Table 1:** Association of the expression levels of three miRNAs with DR severity via regression analysis.

	B	β	t	*p*	F	R
hsa-miR-217	−0.004	−0.325	−2.244	0.028	9.066	0.516
hsa-miR-26a-5p	5.09 × 10^−5^	0.584	4.247	0		
hsa-miR-129-2-3p	−0.003	−0.37	−3.228	0.002		

## Data Availability

All data generated or analyzed during this study are included in this published article.

## Supplementary Materials

The following supporting information can be downloaded at: https://www.mdpi.com/article/10.3390/jcm12051777/s1. Table S1: Expression of RPE-DEGs in our study.
