# Supplementary material for: A 3-miRNA Risk Scoring Signature in Early Diabetic Retinopathy
Source: J Clin Med. 2023 Feb 23;12(5):1777. doi: 10.3390/jcm12051777 (PMC10003264; doi:10.3390/jcm12051777)
Supplement: Supplementary file 1 [file jcm-12-01777-s001.zip › jcm-2121573-supplementary.pdf]

Supplementary Table S1: Expression of RPE-DEGs in our study.

Gene_ID	CON1	CON2	CON3	STZ1	STZ2	STZ3
ENSMUSG00000050966	0	0	0	5.066991532	3.177095115	3.167684897
ENSMUSG00000067578	0	0	0	5.025226421	2.00605213	4.007620291
ENSMUSG00000061356	0	0	0	2.270826485	4.651598376	3.427701512
ENSMUSG00000036480	0	0	2.122148497	4.109400652	3.707884895	3.925752426
ENSMUSG00000024905	0	0	0	2.944013496	3.707884895	2.442727734
ENSMUSG00000037627	0	0	0	3.939930077	2.328382799	2.660842895
ENSMUSG00000090061	3.929590423	0	2.720066038	6.565854296	3.592356107	5.117646015
ENSMUSG00000030607	0	0	0	3.401219228	3.007058354	2.185660269
ENSMUSG00000079042	0	0	1.69403857	4.523528866	1.590343424	3.838957874
ENSMUSG00000044519	3.254401876	2.090899846	0	6.444350553	2.591685525	4.535022392
ENSMUSG00000020323	0	0	0	1.542504516	2.591685525	4.007620291
ENSMUSG00000036815	4.1767582	0	3.141537692	5.914156096	3.32918761	5.979879378
ENSMUSG00000020059	0.962876274	0	0	3.847093261	3.466764276	1.472318664
ENSMUSG00000040478	2.522548064	0	2.720066038	5.551883337	3.177095115	4.295288447
ENSMUSG00000000411	0	0	0	3.26430961	2.328382799	2.185660269
ENSMUSG00000066755	0	2.090899846	0	3.526255002	3.007058354	3.303543136
ENSMUSG00000018341	0	0	0	3.26430961	2.591685525	1.872636564
ENSMUSG00000041567	0	0	0	3.26430961	2.591685525	1.872636564
ENSMUSG00000024215	1.943949927	2.418689046	0	3.526255002	4.651598376	3.74660549
ENSMUSG00000061298	1.943949927	0	0	2.531691868	2.814269573	4.007620291
ENSMUSG00000022212	5.925978104	3.105508201	3.467312551	8.002169064	4.914686402	6.978591143

ENSMUSG00000090206	1.535248838	3.105508201	0	3.939930077	4.592691282	3.427701512
ENSMUSG00000085664	0	0	0	2.531691868	2.591685525	2.185660269
ENSMUSG00000043789	0	0	0	2.531691868	2.591685525	2.185660269
ENSMUSG00000026276	8.966577664	8.639292665	8.508931436	11.36421905	11.11003589	10.94072831
ENSMUSG00000024391	0	0	0	2.270826485	2.328382799	2.660842895
ENSMUSG00000051599	0	2.685617668	0	4.745531419	2.00605213	3.167684897
ENSMUSG00000048652	0	0	0	2.531691868	2.814269573	1.872636564
ENSMUSG00000094584	0	0	0	2.752532989	2.00605213	2.442727734
ENSMUSG00000035592	1.535248838	0	0	2.270826485	3.007058354	3.427701512
ENSMUSG00000026644	0	0	0	2.752532989	1.590343424	2.660842895
ENSMUSG00000036131	0	0	0	1.542504516	3.007058354	2.442727734
ENSMUSG00000091313	0	0	0	1.952145821	2.00605213	3.017690163
ENSMUSG00000042567	0	0	0	1.542504516	1.590343424	3.838957874
ENSMUSG00000028750	2.522548064	4.019165736	3.313601051	5.185488213	5.890570626	5.713299007
ENSMUSG00000042678	2.743116927	0	1.69403857	4.109400652	3.32918761	3.925752426
ENSMUSG00000042641	0	1.666040613	2.122148497	2.944013496	4.708194278	3.017690163
ENSMUSG00000037406	0	0	0	2.531691868	2.814269573	1.472318664
ENSMUSG00000040138	3.254401876	3.4303403	0	5.522163864	3.177095115	4.740538173
ENSMUSG00000031898	1.943949927	0	1.082570403	3.641313149	4.651598376	1.472318664
ENSMUSG00000000794	4.387709518	6.0723085	5.243653708	7.337102697	7.853518965	7.238022669
ENSMUSG00000090675	0	0	0	1.952145821	2.328382799	2.442727734
ENSMUSG00000038255	4.1767582	0	3.467312551	5.581002926	3.32918761	5.386035569

ENSMUSG00000024857	0	2.090899846	0	4.109400652	0	4.589210488
ENSMUSG00000072487	0	0	0	0	3.32918761	3.167684897
ENSMUSG00000046807	5.511342421	6.860360839	5.549362484	7.70432034	8.831413366	7.850626796
ENSMUSG00000092286	2.522548064	0	2.122148497	3.747869053	3.707884895	3.647934497
ENSMUSG00000028730	1.535248838	0	2.451863568	3.26430961	4.007561203	3.167684897
ENSMUSG00000038760	0	0	0	3.113032069	0	3.303543136
ENSMUSG00000028976	0	0	0	1.952145821	2.591685525	1.872636564
ENSMUSG00000031139	1.535248838	0	2.946138632	4.982216099	2.328382799	3.427701512
ENSMUSG00000021335	0	0	0	3.401219228	0	2.850271685
ENSMUSG00000047897	0	0	0	2.944013496	0	3.303543136
ENSMUSG00000044405	0	0	0	2.270826485	2.00605213	1.872636564
ENSMUSG00000045648	3.391206933	2.418689046	0	5.4291455	2.591685525	3.925752426
ENSMUSG00000025127	0	0	0	1.542504516	2.591685525	1.872636564
ENSMUSG00000026650	0	1.666040613	0	4.109400652	0	3.542016358
ENSMUSG00000091705	0	0	0	4.109400652	0	1.872636564
ENSMUSG00000104529	0	0	0	3.526255002	0	2.442727734
ENSMUSG00000005950	2.934392822	1.061231743	3.606212359	4.398246028	4.329589847	4.787647743
ENSMUSG00000095654	0	0	1.082570403	2.531691868	2.591685525	1.872636564
ENSMUSG00000081957	0	1.061231743	0	3.113032069	1.004037573	2.850271685
ENSMUSG00000060509	0	0	0	2.531691868	3.32918761	0
ENSMUSG00000032852	0	0	0	2.531691868	3.32918761	0
ENSMUSG00000038071	0	0	0	3.26430961	2.591685525	0

ENSMUSG00000021499	0	2.090899846	2.122148497	2.944013496	4.095053619	3.017690163
ENSMUSG00000085049	0	0	0	0	3.177095115	2.660842895
ENSMUSG00000049537	0	0	1.082570403	4.462246957	0	2.442727734
ENSMUSG00000031074	0	0	0	2.944013496	0	2.850271685
ENSMUSG00000058063	0	0	0	2.944013496	0	2.850271685
ENSMUSG00000024172	4.387709518	2.090899846	3.957123157	7.086044179	3.592356107	5.531512653
ENSMUSG00000027913	2.934392822	2.418689046	2.122148497	6.106671077	3.707884895	3.427701512
ENSMUSG00000072707	0	0	0	0	2.591685525	3.167684897
ENSMUSG00000032017	0	2.418689046	2.122148497	4.027152357	3.592356107	2.660842895
ENSMUSG00000020279	0	0	0	3.26430961	0	2.442727734
ENSMUSG00000022805	0	0	0	1.952145821	3.707884895	0
ENSMUSG00000091818	0	0	0	0	3.466764276	2.185660269
ENSMUSG00000023935	2.262062774	2.418689046	1.69403857	3.939930077	4.329589847	3.74660549
ENSMUSG00000103897	4.098980837	4.638518268	3.606212359	8.447767474	4.76265352	4.641436727
ENSMUSG00000044071	4.451687601	2.090899846	3.141537692	5.819589778	4.007561203	5.224226784
ENSMUSG00000036872	3.103252005	1.666040613	3.313601051	5.107581509	4.329589847	4.007620291
ENSMUSG00000044749	3.516155501	3.27704726	1.69403857	6.045310129	3.914418281	3.838957874
ENSMUSG00000044499	3.254401876	2.685617668	2.946138632	5.396757127	3.32918761	5.445997906
ENSMUSG00000075224	1.943949927	2.91078525	2.720066038	4.745531419	3.32918761	4.740538173
ENSMUSG00000063260	0	0	0	3.747869053	0	1.472318664
ENSMUSG00000035131	0	1.666040613	1.69403857	4.398246028	0	4.158612521
ENSMUSG00000074991	4.927183216	3.4303403	3.141537692	6.947570864	4.095053619	5.531512653

ENSMUSG00000056145	5.096840502	3.4303403	3.957123157	6.717791143	4.592691282	6.222051065
ENSMUSG00000032259	5.136310975	2.090899846	3.73290578	6.691329973	3.32918761	5.959452065
ENSMUSG00000103310	4.387709518	2.090899846	2.720066038	6.165528337	2.814269573	5.224226784
ENSMUSG00000019892	3.737632382	2.418689046	2.720066038	4.462246957	4.177542066	5.189567415
ENSMUSG00000091530	1.943949927	2.090899846	3.467312551	4.109400652	4.255568098	4.085090858
ENSMUSG00000029123	3.391206933	1.666040613	2.946138632	5.4291455	2.814269573	4.641436727
ENSMUSG00000070471	1.943949927	1.666040613	2.451863568	4.027152357	3.466764276	3.427701512
ENSMUSG00000034780	6.247665056	4.361826063	4.151135438	7.155443879	5.938669383	6.515832464
ENSMUSG00000030092	3.391206933	2.91078525	2.946138632	5.74436118	3.177095115	5.154054848
ENSMUSG00000037771	5.418340148	3.105508201	5.366007904	7.697688635	4.329589847	6.636544121
ENSMUSG00000026527	6.075606355	3.27704726	4.23916087	7.065586675	4.467129932	6.794803576
ENSMUSG00000040373	5.570171827	2.685617668	4.151135438	7.055248102	3.914418281	6.169664286
ENSMUSG00000035262	2.262062774	3.811527364	2.451863568	4.892146383	4.399998326	3.925752426
ENSMUSG00000032034	4.571713829	2.090899846	3.957123157	6.491121765	3.592356107	5.224226784
ENSMUSG00000025576	4.834234417	2.685617668	4.23916087	6.146174827	3.914418281	6.384090841
ENSMUSG00000022667	2.522548064	1.666040613	2.122148497	3.641313149	3.177095115	4.158612521
ENSMUSG00000059361	4.6281788	2.685617668	3.957123157	6.969758848	3.007058354	5.959452065
ENSMUSG00000000317	3.737632382	4.506795319	2.451863568	5.025226421	5.17776549	5.080294503
ENSMUSG00000060284	3.836801677	4.574159616	3.141537692	5.522163864	5.53145101	5.041950197
ENSMUSG00000052026	5.318926901	4.923207835	3.606212359	6.831204098	5.840812808	5.688690365
ENSMUSG00000030790	5.570171827	3.105508201	4.909914901	5.794946883	5.680331537	6.597418486
ENSMUSG00000048540	5.056259714	2.91078525	4.322122925	7.436043414	3.32918761	5.959452065

ENSMUSG00000053797	8.0949651	7.511602155	7.602739159	8.733644033	10.13862727	8.731818842
ENSMUSG00000032313	5.418340148	3.4303403	5.583394214	7.212382723	5.434141611	6.151770601
ENSMUSG00000032564	4.6825168	5.070448176	3.467312551	6.743775704	4.531276166	6.25594776
ENSMUSG00000036510	2.934392822	2.418689046	2.946138632	5.551883337	1.590343424	5.445997906
ENSMUSG00000073530	5.318926901	5.245960311	3.73290578	6.781895557	5.708348945	6.019886259
ENSMUSG00000044162	3.254401876	2.685617668	3.141537692	3.939930077	4.399998326	4.877488855
ENSMUSG00000024987	4.734882271	3.568898378	4.855249662	5.74436118	5.434141611	6.039481216
ENSMUSG00000059832	2.522548064	2.090899846	3.141537692	4.937884026	4.007561203	2.850271685
ENSMUSG00000056895	5.598710727	3.568898378	5.062483225	6.756594562	5.53145101	5.979879378
ENSMUSG00000028661	7.115125378	7.049763561	7.48164725	8.890163658	8.404644797	8.339809052
ENSMUSG00000015484	4.098980837	3.811527364	3.849365952	6.146174827	4.007561203	5.585830282
ENSMUSG00000037548	3.103252005	3.695307466	2.122148497	4.109400652	4.592691282	4.158612521
ENSMUSG00000033080	5.212152946	2.685617668	3.606212359	6.717791143	2.591685525	6.133652191
ENSMUSG00000056215	4.571713829	4.700128069	3.73290578	6.958707511	5.293259892	4.691838216
ENSMUSG00000024112	7.985757116	6.594409515	7.759798643	9.417726264	8.041456186	8.816599858
ENSMUSG00000047976	2.743116927	2.685617668	2.720066038	5.718387257	2.00605213	4.359058811
ENSMUSG00000033007	5.248629387	3.695307466	5.549362484	7.451893405	4.095053619	6.873335776
ENSMUSG00000094626	2.934392822	2.090899846	2.122148497	3.939930077	2.591685525	4.535022392
ENSMUSG00000037143	5.056259714	2.91078525	4.151135438	6.580347242	3.592356107	5.852737774
ENSMUSG00000071719	4.451687601	2.685617668	3.73290578	5.691937119	3.814844301	5.258072993
ENSMUSG00000046110	5.784083813	3.695307466	5.404590497	7.73054646	4.708194278	6.337360783
ENSMUSG00000062257	4.451687601	4.361826063	4.613173581	6.781895557	4.708194278	5.80773917

ENSMUSG00000004933	6.15461996	5.204043174	4.798431246	7.055248102	6.007938225	6.926923142
ENSMUSG00000002341	3.516155501	2.090899846	3.141537692	5.222918698	2.591685525	4.740538173
ENSMUSG00000069227	5.481002964	3.695307466	5.616641636	7.065586675	4.962000765	6.570732522
ENSMUSG00000044164	4.320762058	4.436130831	5.155910474	6.203473478	5.680331537	5.830413907
ENSMUSG00000060212	5.570171827	4.361826063	5.109952997	7.075851688	5.59285884	6.169664286
ENSMUSG00000063687	3.103252005	4.923207835	3.73290578	5.691937119	4.914686402	4.920394936
ENSMUSG00000025318	5.056259714	4.019165736	4.322122925	6.867110119	4.329589847	5.959452065
ENSMUSG00000026220	4.6281788	3.105508201	4.151135438	4.638796168	5.7358326	5.258072993
ENSMUSG00000062995	4.834234417	5.023018355	4.909914901	6.491121765	5.840812808	6.151770601
ENSMUSG00000000560	5.096840502	3.105508201	4.677605819	6.664374383	3.814844301	6.096718111
ENSMUSG00000037568	3.737632382	4.112757281	4.057388167	5.259402562	5.217299892	5.117646015
ENSMUSG00000043008	2.522548064	2.91078525	3.467312551	4.331273552	3.466764276	4.787647743
ENSMUSG00000061393	7.291562147	6.718695694	6.328481459	8.399545303	7.828207258	7.794071434
ENSMUSG00000042515	7.456570812	7.395351639	7.675538502	8.532194358	8.866027542	8.796587002
ENSMUSG00000041771	5.626696014	3.695307466	5.366007904	7.650392997	4.531276166	6.151770601
ENSMUSG00000050822	4.016770272	4.283485491	3.606212359	5.891089269	4.095053619	5.558927086
ENSMUSG00000040629	3.391206933	3.919078847	3.73290578	4.844911115	4.865767557	4.962061748
ENSMUSG00000053519	5.541056967	3.568898378	4.613173581	7.490772885	3.32918761	6.515832464
ENSMUSG00000034570	5.511342421	4.700128069	5.742461147	7.345611913	5.789277377	6.429354643
ENSMUSG00000026786	4.320762058	3.919078847	3.957123157	5.066991532	5.651759231	5.080294503
ENSMUSG00000024530	4.6825168	5.286693865	3.606212359	6.126558159	5.007812562	6.019886259
ENSMUSG00000027419	5.948327369	4.436130831	5.062483225	7.601494168	4.76265352	6.649353705

ENSMUSG00000097638	5.318926901	4.112757281	5.013398232	5.491819271	6.157697734	6.35310625
ENSMUSG00000020088	10.36777096	10.19266576	10.42498874	11.96513799	12.03458539	10.52723723
ENSMUSG00000032507	6.745634183	7.299085076	5.616641636	7.756304326	7.701511987	7.729187663
ENSMUSG00000038530	7.317549001	5.474711645	5.772273277	8.160056169	6.074033042	7.850626796
ENSMUSG00000019888	3.737632382	2.418689046	3.313601051	5.843818804	2.591685525	4.535022392
ENSMUSG00000040867	6.318008447	4.973976077	5.858182544	7.82485514	6.007938225	6.817680924
ENSMUSG00000046321	3.103252005	3.105508201	3.141537692	5.222918698	3.32918761	4.295288447
ENSMUSG00000054226	10.57047862	11.31344632	9.891809898	11.02928266	12.37656882	11.85861031
ENSMUSG00000038665	4.387709518	2.91078525	3.849365952	5.794946883	3.914418281	4.920394936
ENSMUSG00000064065	3.516155501	3.105508201	3.141537692	4.796076816	3.592356107	4.833267522
ENSMUSG00000079157	5.481002964	4.200645406	3.957123157	6.165528337	4.708194278	6.204799306
ENSMUSG00000027270	5.598710727	3.919078847	5.801481814	7.608582008	4.177542066	6.968404516
ENSMUSG00000009394	5.096840502	3.919078847	4.739283051	6.743775704	4.255568098	6.151770601
ENSMUSG00000015981	5.784083813	4.112757281	4.545728659	6.460110036	4.962000765	6.384090841
ENSMUSG00000026424	5.318926901	5.439017403	5.404590497	6.444350553	6.789350486	6.289066286
ENSMUSG00000097886	3.516155501	1.666040613	2.451863568	5.794946883	0	5.189567415
ENSMUSG00000024176	5.450011746	4.700128069	4.322122925	6.691329973	5.25577976	5.852737774
ENSMUSG00000020732	9.662954668	9.477346628	10.16754925	10.84950651	10.81057574	10.97420411
ENSMUSG00000001103	6.335071152	5.899235268	5.68092257	7.737029168	6.651839651	6.851330799
ENSMUSG00000032311	4.016770272	4.574159616	3.73290578	5.637536028	4.815131579	5.189567415
ENSMUSG00000089842	6.43341499	6.268094954	6.016026882	8.762528071	6.434235125	6.82898498
ENSMUSG00000017776	10.59015545	10.540899	11.87701536	12.19412004	12.10609413	12.0099884

ENSMUSG00000036295	6.597954225	5.402417585	5.155910474	7.800302788	5.651759231	6.978591143
ENSMUSG00000027500	7.054606496	5.732328712	5.404590497	7.996776722	5.789277377	7.661247825
ENSMUSG00000096883	4.016770272	3.695307466	4.23916087	5.147060676	4.76265352	5.291143323
ENSMUSG00000074771	3.391206933	4.638518268	4.545728659	5.460822694	5.007812562	5.355093167
ENSMUSG00000074607	5.598710727	5.672107582	5.062483225	7.643508106	5.762802457	6.151770601
ENSMUSG00000027577	5.481002964	7.013820183	6.480696022	7.126108963	7.742735744	7.320425012
ENSMUSG00000010086	5.248629387	4.815975436	4.474975444	7.002413827	4.865767557	5.874721465
ENSMUSG00000050359	7.002159498	6.385260852	5.583394214	7.302554884	8.035939064	6.806287596
ENSMUSG00000045657	4.734882271	5.474711645	5.013398232	6.565854296	5.815275196	6.000021491
ENSMUSG00000043857	4.320762058	4.019165736	4.057388167	6.506381177	3.814844301	5.224226784
ENSMUSG00000048216	4.6825168	4.759214248	4.909914901	6.460110036	5.400189826	5.638176834
ENSMUSG00000082286	10.55781581	9.988669547	11.30717136	11.48371549	11.85394373	11.64818316
ENSMUSG00000027489	4.971504327	3.568898378	4.798431246	5.522163864	5.59285884	5.355093167
ENSMUSG00000042532	5.056259714	3.919078847	4.855249662	6.428417017	4.177542066	6.35310625
ENSMUSG00000009470	10.82944689	10.64345387	10.7266235	12.71021971	10.45720677	12.13194217
ENSMUSG00000034145	5.759016169	5.364865041	5.549362484	7.436043414	5.890570626	6.444132401
ENSMUSG00000019796	6.583747858	6.385260852	8.060900248	8.687531355	7.890672853	7.542672151
ENSMUSG00000033768	7.256168435	8.284475883	7.029734351	9.142960405	8.052427537	8.442731687
ENSMUSG00000066952	4.734882271	4.759214248	4.23916087	5.522163864	5.914820444	5.355093167
ENSMUSG00000054850	4.881457241	5.509544043	5.243653708	5.936859911	6.311742832	6.429354643
ENSMUSG00000028351	5.385957626	4.574159616	4.151135438	6.730841924	4.399998326	6.019886259
ENSMUSG00000004961	4.6281788	3.811527364	3.957123157	6.002903229	4.592691282	4.833267522

ENSMUSG00000009876	5.174730276	5.576783057	5.285602045	6.717791143	5.890570626	6.458760321
ENSMUSG00000021647	7.033855927	7.309005986	7.247014178	8.336908614	8.298057033	7.983015321
ENSMUSG00000004892	7.618631957	7.756016143	6.601864536	8.520968427	8.776229759	7.692526725
ENSMUSG00000029028	10.1257362	10.15603409	8.825631127	8.587049968	8.659095565	8.858571969
ENSMUSG00000098678	8.51742907	7.061548405	8.160075704	7.203047606	6.052336152	7.479517357
ENSMUSG00000036586	2.934392822	7.50298492	3.606212359	3.401219228	4.095053619	3.542016358
ENSMUSG00000050944	4.881457241	4.436130831	5.583394214	3.847093261	4.177542066	3.838957874
ENSMUSG00000043705	8.792216077	8.481563948	9.305059341	7.106215646	8.111322444	8.241719635
ENSMUSG00000049307	8.043990815	7.061548405	8.888231567	6.536424177	7.391720017	6.937405836
ENSMUSG00000042246	6.745634183	6.0723085	6.112411623	5.609546371	4.76265352	5.323472551
ENSMUSG00000036223	5.352831528	4.283485491	4.855249662	3.847093261	3.707884895	3.647934497
ENSMUSG00000074121	6.034415195	6.048819049	5.830110717	4.331273552	4.914686402	5.355093167
ENSMUSG00000009248	5.992013311	5.975964964	6.900715051	4.892146383	5.651759231	5.002558874
ENSMUSG00000010760	5.598710727	4.8705877	4.962584215	4.331273552	3.32918761	4.420129302
ENSMUSG00000055775	7.464345919	5.402417585	6.016026882	5.637536028	4.255568098	5.638176834
ENSMUSG00000073758	7.326108295	7.450171804	7.94788416	6.165528337	6.865975549	6.337360783
ENSMUSG00000055945	5.808723321	5.845716909	4.23916087	4.187211783	4.329589847	4.007620291
ENSMUSG00000057534	6.680374346	5.925267906	6.845693352	5.329713866	4.815131579	5.917708713
ENSMUSG00000070390	6.612022064	6.226825241	5.801481814	4.187211783	5.365419737	5.688690365
ENSMUSG00000066902	4.881457241	4.923207835	5.366007904	4.187211783	3.592356107	3.925752426
ENSMUSG00000082064	5.707535087	5.790136488	5.44216808	4.892146383	3.707884895	4.833267522
ENSMUSG00000031425	10.23430919	10.09713583	7.659675365	7.975003516	8.111322444	8.339809052

ENSMUSG00000032942	7.930696985	7.791831048	7.543463306	6.311664804	6.708426272	6.674636633
ENSMUSG00000037095	6.115654018	5.732328712	7.781949562	5.185488213	6.157697734	4.691838216
ENSMUSG00000034438	6.247665056	4.436130831	5.742461147	4.523528866	4.007561203	4.295288447
ENSMUSG00000017837	10.87682413	10.30722346	10.72185707	8.827756899	10.35678586	9.113250375
ENSMUSG00000066687	8.751490559	9.4685217	9.506949913	7.80648016	8.792680488	7.514944088
ENSMUSG00000036634	7.16368805	6.528006095	2.451863568	3.113032069	4.329589847	5.002558874
ENSMUSG00000091956	4.387709518	3.105508201	5.514508545	3.526255002	2.591685525	3.017690163
ENSMUSG00000027420	5.832949072	9.990211843	7.054209255	6.311664804	6.311742832	6.368681725
ENSMUSG00000024222	10.51634438	10.45750305	11.58530564	9.039242169	10.37000154	9.235092147
ENSMUSG00000028360	4.6281788	5.070448176	5.243653708	4.2610401	3.007058354	3.74660549
ENSMUSG00000033765	4.512948565	3.105508201	4.613173581	1.952145821	3.007058354	3.303543136
ENSMUSG00000006014	5.598710727	4.574159616	4.474975444	2.531691868	4.177542066	3.925752426
ENSMUSG00000056966	4.971504327	5.286693865	3.957123157	2.944013496	3.592356107	3.647934497
ENSMUSG00000026285	4.098980837	3.4303403	3.467312551	1.952145821	2.814269573	2.185660269
ENSMUSG00000035184	8.124706972	6.610543564	7.781949562	7.212382723	6.007938225	5.154054848
ENSMUSG00000019368	6.639752781	5.364865041	6.065024022	5.259402562	4.329589847	4.295288447
ENSMUSG00000028341	7.576207824	6.673333796	6.498641424	5.609546371	6.789350486	4.158612521
ENSMUSG00000037624	5.284206271	3.105508201	3.141537692	2.270826485	2.591685525	2.442727734
ENSMUSG00000103711	5.248629387	5.326308843	5.88571856	3.113032069	4.76265352	4.228568404
ENSMUSG00000066704	9.262513239	6.951849976	9.039930154	6.769300523	6.417360206	7.603177868
ENSMUSG00000025945	0	4.506795319	0	0	0	0
ENSMUSG00000078612	4.250556117	3.811527364	5.200449044	2.752532989	3.814844301	2.185660269

ENSMUSG00000081445	3.737632382	2.91078525	4.057388167	1.542504516	1.590343424	2.850271685
ENSMUSG00000056501	7.376424309	7.887626284	8.125845728	5.959211963	6.890638778	5.80773917
ENSMUSG00000097209	5.248629387	3.811527364	5.155910474	1.952145821	4.095053619	3.427701512
ENSMUSG00000091154	3.836801677	3.105508201	3.313601051	0.968274228	2.591685525	1.872636564
ENSMUSG00000042895	6.569400207	6.544895677	7.407596619	6.066055113	4.708194278	4.920394936
ENSMUSG00000000248	7.456570812	5.543555237	5.858182544	5.491819271	4.467129932	4.007620291
ENSMUSG00000050217	1.943949927	9.738209153	5.88571856	4.398246028	3.592356107	4.641436727
ENSMUSG00000034774	7.291562147	4.973976077	6.831603679	3.113032069	5.25577976	5.761291718
ENSMUSG00000046971	5.626696014	4.574159616	5.583394214	3.939930077	2.328382799	4.535022392
ENSMUSG00000029352	5.654148759	9.658974193	6.803004683	5.491819271	5.434141611	6.096718111
ENSMUSG00000046413	5.014504332	4.361826063	4.400572566	1.542504516	3.592356107	3.542016358
ENSMUSG00000056054	1.943949927	3.105508201	5.858182544	2.944013496	0	2.850271685
ENSMUSG00000063820	4.098980837	4.200645406	4.151135438	2.531691868	3.32918761	1.472318664
ENSMUSG00000033107	4.512948565	3.4303403	3.606212359	1.952145821	2.591685525	1.872636564
ENSMUSG00000030945	5.808723321	4.700128069	4.909914901	4.638796168	2.814269573	2.660842895
ENSMUSG00000052525	5.992013311	5.543555237	4.739283051	3.847093261	2.328382799	4.787647743
ENSMUSG00000029755	5.784083813	3.695307466	5.404590497	2.752532989	2.328382799	4.295288447
ENSMUSG00000046311	10.47171806	8.490309696	9.204775881	7.836976383	6.56256718	8.241719635
ENSMUSG00000076439	5.707535087	5.204043174	2.720066038	3.113032069	2.328382799	2.660842895
ENSMUSG00000045392	4.834234417	2.418689046	3.313601051	1.542504516	2.00605213	1.472318664
ENSMUSG00000050350	1.943949927	0	3.73290578	0	0	0
ENSMUSG00000083288	1.535248838	3.105508201	1.082570403	0	0	0

ENSMUSG00000030671	7.392813762	7.99599712	8.08485642	6.146174827	5.562481626	6.019886259
ENSMUSG00000048230	3.103252005	2.685617668	0	0	0	0
ENSMUSG00000067299	3.103252005	10.18325761	6.065024022	4.109400652	4.329589847	5.041950197
ENSMUSG00000025952	2.743116927	10.61774234	7.029734351	4.462246957	5.17776549	4.787647743
ENSMUSG00000076490	1.943949927	4.019165736	0	0	0	0
ENSMUSG00000075510	0.962876274	2.685617668	2.451863568	0	0	0
ENSMUSG00000076867	2.522548064	3.695307466	0	0	0	0
ENSMUSG00000030775	2.934392822	2.418689046	2.451863568	0	0	1.472318664
ENSMUSG00000060188	4.387709518	1.666040613	5.990889826	0	3.814844301	1.872636564
ENSMUSG00000076752	2.934392822	3.4303403	0	0	0	0
ENSMUSG00000021211	4.6281788	3.4303403	3.73290578	0	3.177095115	2.185660269
ENSMUSG00000085763	2.262062774	2.090899846	2.122148497	0	0	0
ENSMUSG00000082481	5.284206271	3.919078847	3.141537692	1.542504516	1.590343424	2.660842895
ENSMUSG00000073658	4.387709518	11.25306107	7.309345731	5.491819271	5.59285884	5.154054848
ENSMUSG00000048655	2.934392822	1.666040613	2.122148497	0	0	0
ENSMUSG00000046160	4.571713829	5.326308843	2.122148497	2.270826485	0	2.850271685
ENSMUSG00000049653	3.254401876	3.811527364	3.957123157	2.531691868	1.590343424	0
ENSMUSG00000100254	0	7.001637266	0	0	0	0
ENSMUSG00000110533	2.743116927	3.4303403	2.451863568	0	1.590343424	0
ENSMUSG00000095438	1.535248838	2.685617668	2.946138632	0	0	0
ENSMUSG00000092746	9.530350632	0	0	0	2.328382799	0
ENSMUSG00000099250	13.9482711	5.116368207	5.109952997	5.914156096	5.7358326	5.224226784

ENSMUSG00000099021	13.93687738	5.070448176	5.200449044	5.74436118	5.762802457	5.224226784
ENSMUSG00000060180	5.626696014	4.361826063	4.909914901	2.944013496	1.590343424	2.850271685
ENSMUSG00000065037	12.36980068	4.638518268	4.474975444	5.066991532	4.531276166	4.007620291
ENSMUSG00000061531	1.943949927	3.919078847	2.122148497	0	0	0
ENSMUSG00000051900	1.943949927	2.685617668	3.467312551	0	0	0
ENSMUSG00000029417	2.262062774	4.436130831	3.957123157	2.531691868	0	0
ENSMUSG00000103081	6.22952992	11.77546516	6.287779049	5.664992978	5.007812562	5.041950197
ENSMUSG00000108487	2.522548064	4.283485491	4.057388167	0	0	2.185660269
ENSMUSG00000004709	3.391206933	3.919078847	3.467312551	0	0	1.872636564
ENSMUSG00000048582	2.522548064	4.8705877	2.122148497	0	0	0
ENSMUSG00000032401	2.522548064	7.152514093	4.739283051	0	2.591685525	2.185660269
ENSMUSG00000032517	10.44642496	10.63263178	6.713640189	1.952145821	5.815275196	7.883525535
ENSMUSG00000064365	6.335071152	6.510916444	5.285602045	0.968274228	1.004037573	0.916352336
